# Affective Priming by Simple Geometric Shapes: Evidence from Event-related Brain Potentials

**DOI:** 10.3389/fpsyg.2016.00917

**Published:** 2016-06-17

**Authors:** Yinan Wang, Qin Zhang

**Affiliations:** Beijing Key Laboratory of Learning and Cognition, Department of Psychology, Capital Normal UniversityBeijing, China

**Keywords:** circle, downward triangle, emotional meaning, affective priming paradigm, event-related potential (ERP), LPP

## Abstract

Previous work has demonstrated that simple geometric shapes may convey emotional meaning using various experimental paradigms. However, whether affective meaning of simple geometric shapes can be automatically activated and influence the evaluations of subsequent stimulus is still unclear. Thus the present study employed an affective priming paradigm to investigate whether and how two geometric shapes (circle vs. downward triangle) impact on the affective processing of subsequently presented faces (Experiment 1) and words (Experiment 2). At behavioral level, no significant effect of affective congruency was found. However, ERP results in Experiment 1 and 2 showed a typical effect of affective congruency. The LPP elicited by affectively incongruent trials was larger compared to congruent trials. Our results provide support for the notion that downward triangle is perceived as negative and circle as positive and their emotional meaning can be activated automatically and then exert an influence on the electrophysiological processing of subsequent stimuli. The lack of significant congruent effect in behavioral measures and the inversed N400 congruent effect might reveal that the affective meaning of geometric shapes is weak because they are just abstract threatening cues rather than real threat. In addition, because no male participants are included in the present study, our findings are limited to females.

## Introduction

Simple geometric patterns might convey emotional meaning. For example, diagonal and angular configurations tend to be associated with threat, while rounded features and curved lines tend to be linked to pleasantness and happiness. That association is evident in a wide array of observations, including facial features of tribal masks (Aronoff et al., 1988), preference for babyish faces and the caregiving for infants that may derive from their rounded facial features (Zebrowitz, 1997), and the configurations of human bodies in 17th-century Dutch art (Aronoff, 2006).

In addition to those observations, subjective evaluations in a few studies have demonstrated the emotional meaning of simple geometric shapes. Participants were asked to evaluate some geometric patterns on a set of subjective semantic differential scales (Osgood et al., 1957) and indicated the degree of “badness”, “potency” and “activity” of each visual stimulus. It turned out that the sharp-v and rounded images conveyed an angry and a happy meaning, respectively (Aronoff et al., 1988, 1992). Bar and Neta (2006) obtained similar findings in a two-choice like/dislike forced choice task. Participants disliked neutral objects comprised of pointed features and sharp angles significantly more than curved ones (e.g., a watch with a sharp-angled contour in comparison with curved contour). Pavlova et al. (2005) also found a significant positive correlation between rated negative emotions and perceived instability of geometric shapes such as the triangle and the oval. Apart from those explicit subjective evaluations, Larson et al. (2012) used the Implicit Association Test to examine implicit attitudes toward circles and toward downward- and upward-pointing triangles. The results showed that participants were faster to categorize downward triangles as unpleasant, rather than neutral or pleasant, suggesting that people can extract affective meaning from simple geometric shapes and this affective perception could occur even without explicit affective value judgments.

Consistent with findings that stimuli that signal potential threat could capture attention preferentially, v-shapes could be detected faster than some other shapes for their threat-related implications. Tipples et al. (2002) found faster detection of target faces (schematic face or the outline of a face) with threatening v-shaped eyebrows compared to non-threatening Λ-shaped eyebrows, suggesting that a v-shape played an important role in conveying negative meanings. Larson et al. (2007), using a visual search task, found that participants detected v-shapes or downward triangles faster than inverted v-shapes or triangles, when those shapes were embedded in a field of other shapes. Moreover, Larson and colleagues demonstrated that, in some cases, during trials of homogeneous fields of stimuli, fields of v-shapes led to slower response times, suggesting that this shape could cause difficulty in disengaging attention. Further evidence provided by Watson et al. (2011) showed that, when displays contained a varying number of elements and in which the stimuli were randomly arranged, searching for a downward triangle among upward triangles still was more efficient than the reverse. Furthermore, a search advantage for the downward-pointing triangle remained, regardless of whether the search display was presented within the context of a floor or ceiling perspective.

Processing of threatening stimuli such as v-shaped configuration or downward triangles also induced activation in the amygdala in several functional magnetic resonance imaging (fMRI) studies. For instance, the results of Bar and Neta (2007) revealed that neutral objects containing sharp features could activate the amygdala as compared to curved contours. Because previous studies (e.g., Vuilleumier et al., 2003; Whalen et al., 2004) showed that the amygdala exerted a particularly important influence on the detection of threat, the findings from Bar and Neta (2007) developed the idea further that sharp features can help signal a potential danger. Moreover, Larson et al. (2009) found that simple shapes with a downward v feature elicited greater activation of the amygdala and other neural networks (e.g., subgenual anterior cingulate cortex, fusiform gyrus, and superior temporal gyrus, and extrastriate visual regions). Those findings confirmed that simple v-shapes which were devoid of contextual affective cues could act like common emotional stimuli (such as facial expression) and activate the same neural circuitry known to process realistic, contextual threatening stimuli. Apart from fMRI evidence, recent research (Armbruster et al., 2014) investigated the effects of circles, as well as upward- and downward-point triangles, on three peripheral physiological markers: skin conductance response (SCR), startle reflex, and facial electromyography (EMG). In the first study, Armbruster et al. (2014) administered acoustic startle probes alone and during viewing of simple geometric shapes. In the second study, participants viewed the same geometric shapes, while measuring skin conductance on the non-dominant left hand and EMG over zygomaticus major and corrugators supercilii muscles. Armbruster et al. (2014) found that the presentation of circles resulted in significant reductions of startle and SCR magnitudes, compared to upward and downward pointing triangles, demonstrating that circle might be the most pleasant and least arousing among the three geometric shapes. In addition, Pavlova et al. (2010a) identified the cortical network engaged in visual processing of social interaction revealed by the motion of simple geometric shapes by analysis of oscillatory gamma magnetoencephalographic (MEG) activity. They found that the right posterior temporal cortex is a key region for the networks engaged in visual perception of social interaction. Moerover, a gender difference in the induced gamma response was found over the left prefrontal cortex, a perceptual decision-making region (Pavlova et al., 2010b).

It is worth noting that simple geometric shapes were task-relevant stimuli in the above-mentioned experimental studies and in most of these studies participants were asked to explicitly respond to the shapes. What would happen, if these shapes are task-irrelevant stimuli and used only as backgrounds? Although many previous studies have explored emotional context effects (e.g., Van den Stock and de Gelder, 2014; Van den Stock et al., 2014a,b; Zhang et al., 2015), only a few studies have focused on effects of geometric shape context. In one study, Toet and Tak (2013) asked participants to judge perceived facial dominance of neutral faces presented on downward or upward background triangles. They found that participants judged neutral faces as more dominant on a downward triangle background, compared to an upward one. Other research (Watson et al., 2012) used the flanker task with simple geometric shapes as flanker stimuli. Participants evaluated the central face. The result showed that downward triangles interfered with valence judgments for the central target in a similar way to a negative face flanker: Responses were faster to negative face targets, but slower to positive ones, providing converging evidence that a downward triangle conveys negative valence.

The present study further examined effects of geometric shape context. In contrast to the studies of Watson et al. (2012) and Toet and Tak (2013), in which participants viewed the target stimulus and background shape simultaneously, the current study sequentially presented background and target to participants. More specifically, the present study employed the affective priming procedure in which participants viewed two affective stimuli in sequence and then evaluated the second stimulus (the target), without having to respond to the first one (the prime or context). The congruent effect occurs, when affectively congruent trials (i.e., a positive prime followed by a positive target or a negative prime followed by a negative target) lead to faster and less error-prone responses, compared to affectively incongruent trials (i.e., positive prime – negative target or negative prime – positive target). Because no response for primes was required, participants would process those shapes automatically. Thus, this procedure allows for investigation of whether the emotional meaning of different background shapes could be activated without active processing and then influence the participants’ responses to targets. Our first experiment used downward triangles and circles as primes and used emotional faces as targets to investigate the effects of simple geometric forms on the evaluation of different emotional faces. In order to rule out the influence of the similarity between the circle and the facial contour on affective priming, the second experiment used the same prime stimuli, but with emotional words as targets. On the basis of substantive evidence from the literature that simple geometric shapes were associated with different emotional meanings, we expected that the affective meaning of these shapes could be automatically activated and then influence the evaluation of targets in a consistent manner. In other words, performance measured in terms of accuracy or reaction time (RT) would be better on congruent trials (downward triangle-negative target; circle-positive target) than on incongruent ones (downward triangle-positive target; circle-negative target).

In addition to behavioral measures, we also assessed event-related potential (ERP) measures to investigate the electrophysiological correlates of affective processing in the affective priming paradigm. Such electrophysiological indicators can reflect a temporally precise stream of neural activity from the moment a stimulus is presented until after the response is executed (Herring et al., 2011). We would concentrate on the N400 component and the late positive potential (LPP) based on previous affective priming studies (e.g., Werheid et al., 2005; Zhang et al., 2010, 2012). The N400 component is a negative deflection that generally peaks around 400ms after target onset. Although many studies (see Kutas and Federmeier, 2011, for a recent review) have shown that its amplitude relies on the semantic relationship between the target and the context in which the target appears, N400 effects have also been found in the affective priming paradigm. Much research (e.g., Zhang et al., 2006, 2010; Krombholz et al., 2007; Eder et al., 2011) has demonstrated that affective mismatches between primes and targets evoked a larger N400 response than congruent pairs. LPP which appears in a time window between 400 and 700 ms is a positive component that is sensitive to the affective or motivational value of the stimuli. There is additional evidence in affective priming studies showing that evaluatively incongruent stimuli led to larger LPP amplitudes in comparison to evaluatively congruent ones, when participants categorize targets as positive or negative (Werheid et al., 2005; Zhang et al., 2010; Herring et al., 2011). In addition, we also examined the N170 component because different expressions would be used as targets in the present experiment. N170 is regarded as a face sensitive potential, which is indicated by a negative waveform in the 120–220ms range with an average latency of 170 ms (Bentin et al., 1996). Many studies (e.g., Batty and Taylor, 2003; Eger et al., 2003; Blau et al., 2007; Leppänen et al., 2007; Japee et al., 2009) reported that facial emotional expression of real and morphed faces affected the face sensitive N170. For example, Hietanen and Astikainen (2013) recently showed a clear affective priming effect on this face sensitive component: N170 amplitudes to happy faces were larger when presented after positive than negative primes, whereas the N170 amplitudes to sad faces were larger when presented after negative than positive primes.

To sum up, the current study measured behavioral and electroencephalography (EEG) activity, applying an affective priming procedure that has been widely used as a tool to measure the effects of automatic evaluation, with downward triangles and circles as primes and expressions (Experiment 1) or words (Experiment 2) as targets. At behavioral level, we expected that affective priming should manifest as greater RTs and lower accuracy on affectively incongruent trials than on congruent trials. In electrophysiological terms, we focused our interest on the N170 at the P7 and P8 electrode sites (Experiment 1), as well as the N400 and LPP components on a wide range of brain areas (frontal, fronto-central, centro-parietal, and parietal) in both two experiments. According to the ERP studies mentioned above, we expected the N170 amplitudes to be larger for targets preceded by affectively congruent primes compared to incongruent primes. With respect to the N400 and LPP, we expected that the amplitudes would be greater in affectively incongruent prime-target trials than in affectively congruent prime-target trials. In addition, because previous studies (De Houwer et al., 2001; Werheid et al., 2005; Zhang et al., 2010; Hietanen and Astikainen, 2013; Okubo and Ogawa, 2013) have shown that the RT to positive facial expressions or words were significantly faster than to negative facial expressions or words in the affective priming procedure, we also manipulated target valence (positive vs. negative) to examine whether this factor could impact affective priming effect.

## Materials and Methods

### Participants

Nineteen right-handed students (aged 19–24 years, mean age = 20.89 years, *SD* = 1.41 years) from Capital Normal University took part in two experiments. Only women were recruited in this study for ruling out the gender differences in emotion processing (Kring and Gordon, 1998; Kret and De Gelder, 2012). All participants had normal or corrected-to-normal vision. Informed consent was obtained from each participant and all participants were financially compensated for their involvement. This study was approved by the Institutional Review Board of the Capital Normal University.

### Materials

In each of two experiments, the stimuli consisted of 80 prime-target pairs, which were divided into two groups: 40 prime-target pairs using circles (○) as primes and 40 prime-target pairs using downward triangles (▽) as primes. In addition, there were 40 filler pairs (squares as primes). Each of 120 pairs was repeatedly presented three times in each experiment. Assuming a viewing distance of 60 cm, the visual angles were 3.82 × 3.30° for downward triangle, 3.82° in diameter for circle, and 3.82 × 3.82° for squares. Another nineteen undergraduates who didn’t participate in the two experiments evaluated each geometric shape on four 7-point scales (Lundqvist et al., 1999, 2004; Lundqvist and Öhman, 2005) that were labeled from -3 (negative valence) to +3 (positive valence): Good–Bad, Kind–Cruel, Friendly–Unfriendly and Pleasant–Unpleasant. The valence of each shape was calculated as the mean of the four scales. The result showed that the average valence of the three shapes were 1.67, -0.61, and 0.97 for circle, downward triangle, and square, respectively. The one-way repeated measures analysis of variance (ANOVA) revealed a main effect of valence, *F*(2,56) = 17.69, *p* < 0.001. Further analysis revealed that there were reliable differences on valence between circle and downward triangle (*p* < 0.001), as well as between square and downward triangle (*p* < 0.001). And the difference between circle and square reached a marginal significance (*p* = 0.081). These results indicated that circle was rated more positively than downward triangle on subjective judgments.

In Experiment 1, target stimuli were 40 pictures of faces (10 women and 10 men faces, 20 angry faces and 20 happy faces) selected from Chinese Facial Affective Picture System (Wang and Luo, 2005). The mean valence (a continuum ranging from unpleasant to pleasant) on a 1–9 point scale (with 9 being the most positive in valence dimension) was 2.88 for angry faces and 6.50 for happy faces, respectively. The mean arousal (a continuum ranging from calm to excited) on a 1–9 point scale (with 9 being the highest arousal) was 5.88 for angry faces and 5.12 for happy faces, respectively. There was no significant difference in arousal level between angry and happy faces (*p* > 0.05) but the valence difference between those two reached significance (*p* < 0.01). In order to make 80 prime-target pairs (40 affectively congruent pairs and 40 affectively incongruent pairs) and 40 filler pairs, each target was used in three separate pairs.

In Experiment 2, target stimuli were 20 negative words and 20 positive words which were chosen from the Chinese Affective Words System (Luo and Wang, 2004). The mean valence on a 1–9 point scale (with 9 being the most pleasant) was 2.98 for negative words and 6.70 for positive words, respectively. The mean familiarity (indicating how familiar people are with different words) on a 1–9 point scale (with 9 being the most familiar) was 5.41 for negative words and 5.34 for positive words, respectively. There was no significant difference in familiarity between negative and positive words (*p* > 0.05) but the valence difference between those two reached significance (*p* < 0.01).

### Procedure

**FIGURE 1:**
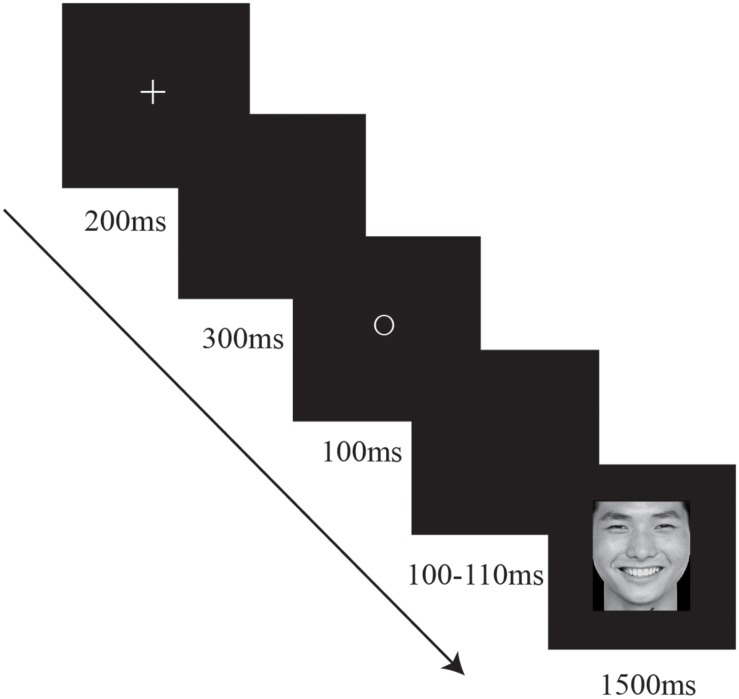
**Example event sequences on a prime-target trial in Experiment 1**.

Stimuli were presented against a black background in the center of a 17″CRT monitor (1024 × 768 resolution, 100-Hz refresh rate). Each trial was started by a presentation of fixation cross “+” for 200 ms followed by a 300 ms blank screen (see **Figure 1**). Next, one of the three geometric shapes appeared for 100 ms followed by a blank screen ranging from 100 to 110 ms randomly. A target was then presented for 1500 ms. Participants were instructed to categorize each face as angry or happy as quickly and accurately as possible in Experiment 1. And they were instructed to categorize each word as negative or positive in Experiment 2. Responses were entered via mouse key and the assignment of the left and right hand to target response was counterbalanced across participants. Besides, each participant received three identical blocks in each experiment. The stimulus pairs were presented with random order in each block which consisted of 120 trials. Prior to testing, each participant performed 12 practice trials to ensure that the procedure was well understood.

### EEG Recordings and Data Processing

Electroencephalogram (EEG) was continuously recorded from 64 scalp electrodes using an electrodes cap with Ag/AgCl inserts. A left mastoid reference electrode was used online and the reference was changed offline to the average of left and right mastoid recordings. Vertical and horizontal electrooculograms (EOG) were recorded with two pairs of electrodes, one pair placed above and below the left eye, and another pair placed beside the two eyes. EEG signals were filtered with a band-pass from 0.05 to 40 Hz and sampled at a rate of 500 Hz. Electrode impedances were kept below 5 KΩ. Average ERPs were formed offline from correct-response trials. Epochs containing artifacts exceeding ±75 μV were excluded from ERP analyses. Each averaging epoch lasted 1200 ms, including 400 ms prior to target onset.

The present study mainly focused on three ERP components: N170, N400 and LPP. The amplitude measurements were referred to pre-prime baseline (-400 to -210 ms). Based on visual inspection of our waveform and a review of previous finding, mean amplitudes were calculated at time windows 130–210 ms (N170), 310–410 ms (N400), and 450–650 ms (LPP for Experiment 1) or 410–650 ms (LPP for Experiment 2). The N170 was measured at the P7 and P8 electrode sites and a 2(affective congruency: congruent, incongruent) × 2(target valence: angry, happy) × 2(electrode location: P7, P8) repeated-measures ANOVA was conducted for N170. Regarding N400 and LPP, the ANOVAs were conducted by selecting 24 electrodes from left hemisphere and right hemisphere at anterior locations (frontal and fronto-central) and posterior locations (centro-parietal and parietal). These electrodes were divided into four areas: Left-Anterior (LA: F1, F3, F5, FC1, FC3, F5), Left-Posterior (LP: P1, P3, P5, CP1, CP3, CP5), Right-Anterior (RA: F2, F4, F6, FC2, FC4, FC6), and Right-Posterior (RP: P2, P4, P6, CP2, CP4, CP6). The mean amplitude for each of the four areas under each condition was computed. Two congruency (congruent, incongruent) × 2 target valence (angry/negative, happy/positive) × 2 laterality (left, right) × 2 location (anterior, posterior) repeated-measures ANOVAs were performed for N400 and LPP components.

## Results

Each participant’s mean recognition accuracies and RTs to target faces (Experiment 1) or words (Experiment 2) were computed. Data beyond three standard deviations of the mean value were discarded from further analyses (2% for both Experiments 1 and 2). The Shapiro–Wilk test for normality showed that RT data were normally distributed (Ws > 0.91, *p*s > 0.05).

### Experiment 1

**Table 1** displays mean RTs and accuracy rates under affectively congruent and incongruent conditions in Experiment 1.

**Table 1:** Mean RTs (ms) and accuracies to angry and happy faces under different priming conditions (M ± SD) in Experiment 1.

	Angry faces		Happy faces	
		
	Incongruent	Congruent	Incongruent	Congruent
RT	616 ± 87	626 ± 88	597 ± 93	590 ± 90
Accuracy	0.97 ± 0.03	0.97 ± 0.03	0.99 ± 0.02	0.98 ± 0.02


**FIGURE 2:**
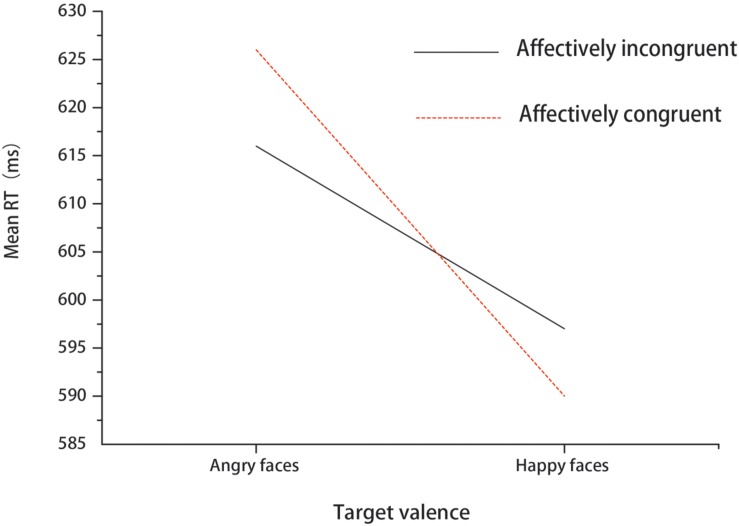
**Mean RT for different expressions under affectively incongruent or congruent primes in Experiment 1**.

A 2 (affective congruency: congruent, incongruent) × 2 (target valence: angry, happy) repeated-measures ANOVA was performed on RTs. The main effect of congruency was not significant [*F*(1,18) = 0.19, *p* > 0.05, $\eta_{p}^{2}$ = 0.11]. The main effect of target valence was significant [*F*(1,18) = 21.94, *p* < 0.001, $\eta_{p}^{2}$ = 0.55, 1-β = 0.99], with slower responses when angry faces were presented compared to happy faces. The interaction between congruency and target valence also reached significance [*F*(1,18) = 7.47, *p* < 0.05, $\eta_{p}^{2}$ = 0.29, 1-β = 0.73] (see **Figure 2**). Simple effect analysis showed that participants responded to angry faces slower in congruent trials than incongruent trials (*p* < 0.05). Additionally, the two-way repeated-measures ANOVA for accuracies did not find any significant effects in Experiment 1 (*ps* > 0.05).

**FIGURE 3:**
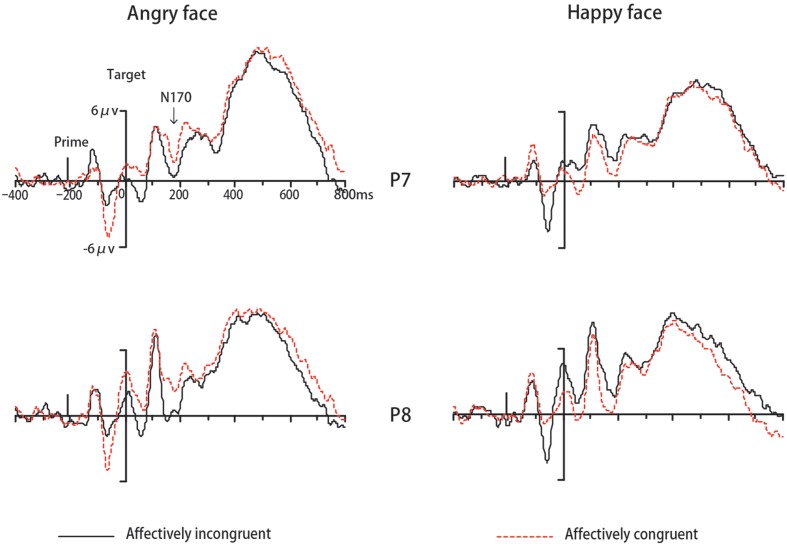
**Grand mean ERPs to different expressions from P7and P8 under affectively incongruent or congruent primes in Experiment 1**.

**FIGURE 4:**
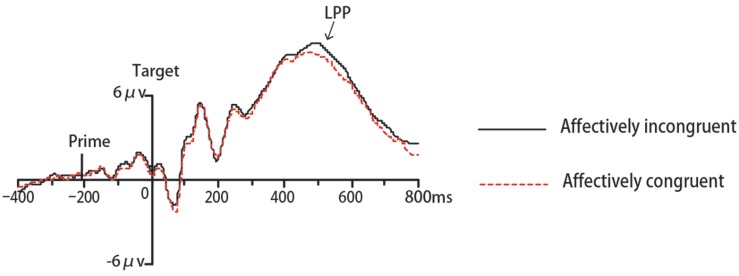
**Grand mean ERPs to expressions from POz under affectively incongruent or congruent primes in Experiment 1**.

In Experiment 1, the ERP data of eighteen participants were remained for analyses while one was excluded due to excessive ocular artifacts and electrode drift (>25% of trials). The ANOVA for N170 revealed a significant interaction between congruency and target valence [*F*(1,17) = 16.42, *p* < 0.01, $\eta_{p}^{2}$ = 0.49, 1-β = 0.97]. Simple effect analysis indicated that amplitudes for angry faces were more negative-going in incongruent trials than congruent trials (*p* < 0.001). On the contrary for happy faces, N170 amplitudes were more negative-going in congruent trials than incongruent trials (*p* < 0.001) (see **Figure 3**). No other main effects or interactions were significant (*p*s > 0.05). The analysis for N400 did not find any significant effects (*ps* > 0.05). Regarding LPP, there was a significant main effect of location [*F*(1,17) = 6.56, *p* < 0.05, $\eta_{p}^{2}$ = 0.28, 1-β = 0.68], with more positive-going amplitude on posterior areas compared to anterior areas. The main effect of target valence also reached significance [*F*(1,17) = 9.71, *p* < 0.01, $\eta_{p}^{2}$ = 0.63,1-β = 0.84], due to more positive-going ERP amplitude for angry faces than happy faces. The interaction between location and target valence was significant [*F*(1,17) = 4.76, *p* < 0.05, $\eta_{p}^{2}$ = 0.22, 1-β = 0.54]. No other main effects or interactions were significant (*p*s > 0.05). Then we performed an additional 2 congruency (congruent, incongruent) × 2 target valence (angry, happy) repeated-measures ANOVA for LPP amplitude at the parietal-occipital site on midline (POz) based on typical scalp distributions of LPP reported by previous studies (Lu et al., 2010; Herring et al., 2011) and our visual inspection of the grand average waveforms. There was a significant main effect of congruency [*F*(1,17) = 5.65, *p* < 0.05, $\eta_{p}^{2}$ = 0.25, 1-β = 0.61], due to more positive-going ERP amplitude in incongruent trials than congruent trials (see **Figure 4**). A significant effect of target valence was also found [*F*(1,17) = 14.68, *p* < 0.01, $\eta_{p}^{2}$ = 0.46, 1-β = 0.95], with more positive-going ERP amplitude for angry faces than happy faces. The interaction between valence and target valance was not significant (*p*s > 0.05).

### Experiment 2

**Table 2** displays mean RTs and accuracy rates under affectively congruent and incongruent conditions in Experiment 2.

**Table 2:** Mean RTs (ms) and accuracies to negative and positive words under different priming conditions (M ± SD) in Experiment 2.

	Negative words		Positive words	
		
	Incongruent	Congruent	Incongruent	Congruent
RT	606 ± 85	616 ± 96	581 ± 95	579 ± 83
Accuracy	0.97 ± 0.04	0.98 ± 0.03	0.98 ± 0.02	0.97 ± 0.04


A 2 (affective congruency: congruent, incongruent) × 2 (target valence: negative, positive) repeated-measures ANOVA was performed on RTs. The main effect of congruency was not significant [*F*(1,18) = 1.64, *p* > 0.05, $\eta_{p}^{2}$ = 0.08]. The main effect of target valence was significant [*F*(1,18) = 35.20, *p* < 0.001, $\eta_{p}^{2}$ = 0.66, 1-β = 1], with slower responses when negative words were presented compared to positive ones. The interaction between congruency and target valence did not reach significance [*F*(1,18) = 1.41, *p* > 0.05, $\eta_{p}^{2}$ = 0.29]. Additionally, the two-way repeated-measures ANOVA for accuracies did not find any significant effects in Experiment 2 (*ps* > 0.05).

**FIGURE 5:**
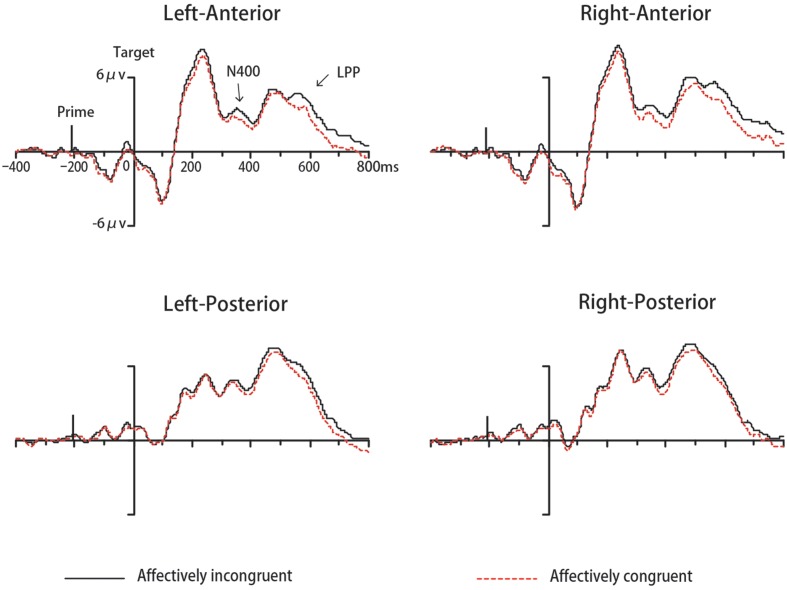
**Grand mean ERPs to words from four areas under affectively incongruent or congruent primes in Experiment 2**.

The ERP data of seventeen participants were remained for analyses in Experiment 2 while two participants were excluded. The analysis for N400 indicated a significant main effect of congruency [*F*(1,16) = 4.64, *p* < 0.05, $\eta_{p}^{2}$ = 0.23, 1-β = 0.53]. The average amplitude was significantly more negative-going to a target that was affectively congruent with the preceding prime compared to incongruent one (See **Figure 5**). The main effect of location also reached significance [*F*(1,16) = 8.82, *p* < 0.05, $\eta_{p}^{2}$ = 0.36, 1-β = 0.80], with more negative-going amplitude on anterior areas compared to posterior areas. No other main effects or interactions were significant (*p*s > 0.05). The analysis for LPP indicated a significant effect of congruency [*F*(1,16) = 7.46, *p* < 0.05, $\eta_{p}^{2}$ = 0.32, 1-β = 0.73], with incongruent trials elicited larger LPP amplitudes than congruent trials (See **Figure 5**). The main effect of location also reached significance [*F*(1,16) = 7.12, *p* < 0.05, $\eta_{p}^{2}$ = 0.31, 1-β = 0.71], with more positive-going amplitude on posterior areas compared to anterior areas. The main effect of target valence was observed [*F*(1,16) = 6.72, *p* < 005, $\eta_{p}^{2}$ = 0.30, 1-β = 0.68], with more positive-going amplitudes elicited by negative words compared to positive words. No other main effects or interactions were significant (*p*s > 0.05)

## Discussion

Previous work has demonstrated that simple geometric shapes may convey emotional meaning using various experimental paradigms. However, it has been unclear until now whether the emotional meaning of shapes could be activated without active processing and what the impact on the responses to a subsequent stimulus would be. Therefore, by employing an affective priming paradigm, we aimed to investigate whether the emotional meanings of circle and downward triangle could be activated automatically and then influence participants’ responses to targets. We conducted two similar experiments, using same shapes (circle and downward triangle) as primes and either facial expressions (Experiment 1) or emotional words (Experiment 2) as targets.

The behavioral result of Experiment 1 showed a significant interaction between affective congruency and target valence. The responses to angry faces were faster after the presentation of incongruent primes than after congruent primes, which contradicted our expectation. We suppose that the structural similarity between circle and facial contour may have contributed to the experimental results. Previous work (Aronoff et al., 1988, 1992; Bar and Neta, 2006) and the subjective evaluation results of the present study all showed that the circle tends to be evaluated as positive, but the downward triangle tends to be evaluated as negative. Therefore, when angry faces were used as targets, prime stimuli were supposed to be circles in affectively incongruent trials and downward triangle in congruent trials according to those previous work and evaluation results. However, circle prime – angry face pair might be perceived as more congruent in structure than triangle prime – angry face pair. The structural similarity between the circle prime and the angry facial contour might have facilitated the responses to angry faces to some extent, resulting in faster evaluation in affectively incongruent trials than in congruent trials. When words were used as targets in the second experiment, the structural similarity between circle prime and target was ruled out. The unexpected congruency effect (i.e., faster responses in affectively incongruent trials than congruent ones) vanished. Additionally, the results of Experiment 1 did not indicate a facilitation effect of the circle on responses to happy faces. A possible explanation is that RTs to happy faces reached floor levels.

On the behavioral level, neither Experiment 1 nor 2 showed a significant affective priming effect in the expected direction. There might be two reasons for that outcome. On the one hand, the present evaluation task may not be sensitive enough to pick up subtle behavioral effects. In fact, such an affective congruency effect has not always been found in previous studies (Klauer and Musch, 2001; Storbeck and Robinson, 2004; Aguado et al., 2013) and several researchers have reported affective priming effects on the electrophysiological level in the absence of significant behavioral priming results (Hinojosa et al., 2009; Kissler and Koessler, 2011). On the other hand, compared to facial expressions or scenes with emotional meanings, simple geometric forms are more abstract and potentially weaker affective cues. When simple shapes were used as primes, the weak evaluative or response bias caused by them cannot effectively facilitate or inhibit the selection of the correct responses during the evaluation for targets. As a result, we did not observe a significant affective priming effect in the RT and accuracy rate data. That is, the affective meaning of simple geometric shapes may be not strong enough to influence the evaluation of the targets on a behavioral level. In fact, the subjective evaluation results showed that the downward triangle was only slightly negative, with a mean valence of -0.61 on a 7-point scale from -3 (negative valence) to +3 (positive valence), supporting our explanation. Additionally, Armbruster et al. (2014) measured the influence of geometric shapes on peripheral physiological markers. Their results did not reveal a significant effect of geometric forms on facial EMG and there was also no significant difference between downward and upward triangles in SCR and startle magnitudes. Armbruster et al. (2014) concluded that, although the underlying neuronal pattern activated by downward triangles may be similar to that activated by real stimuli (such as angry faces), it was still necessary for individuals to differentiate between realistic threat stimuli and abstract geometric threat cues. This mechanism to discriminate was particularly important for individuals in order to facilitate appropriate responses and avoid unnecessary costly reactions (Armbruster et al., 2014). In the present study, the absence of a behavioral priming effect may reflect that participants were enabled to automatically differentiate between real affective stimuli and abstract affective cues (simple shapes).

In addition, our behavioral data showed that participants’ responses to negative targets (angry expressions or negative words) were slower than those to positive targets (happy expressions or positive words). Such an effect had been reported in previous affective priming studies, either with expression targets (Werheid et al., 2005; Hietanen and Astikainen, 2013) or word targets (De Houwer et al., 2001; Zhang et al., 2010; Okubo and Ogawa, 2013). The effect probably suggests there is greater difficulty in disengaging attention from a negative stimulus than from a positive stimulus (Larson et al., 2007). Further investigations will be required to clarify this issue.

Compared to behavioral measures, ERP measurements can provide additional information. As we described in the Introduction, components of the ERP are known to be related to certain mental processes. The present study followed N170, N400, and LPP components with interest. According to our ERP data, the amplitudes of N170 showed opposite patterns during the evaluation of angry and happy faces, under two different priming conditions. More specifically, the N170 response to happy faces was more negative-going after affectively congruent primes (circles) than after incongruent primes (downward triangle), while the N170 elicited by angry faces was more positive-going after affectively congruent primes (downward triangle) than after affectively incongruent primes (circles). In other words, the N170 was more negative in the circle prime condition than in the downward triangle prime condition. Therefore, we thought that the present result reflected the effect of the priming type. In contrast with our results, Hietanen and Astikainen (2013), using an affective priming paradigm with natural scenes (prime) and expressions (target) as stimuli, found that the N170 amplitudes elicited by facial expressions were more negative-going in affectively congruent trials than in incongruent trials. Hietanen and Astikainen (2013) argued that the N170 priming effect reflected the integration of activation elicited by the scenes and faces in specific brain regions. However, our Experiment 1 showed a priming type effect, but not an affectively congruent effect. Similar to the explanation of RT result, we think that the similarity between circle and face contour might play an important role in the present N170 effect. Considering that the N170 component reflects the visual processing of configurally represented information in the face (Bentin et al., 1996; Eimer, 2000; Sagiv and Bentin, 2001), it seems reasonable to suppose that it would be susceptible to the structural similarity with the stimuli used in the present study. Because the priming effect produced by this structural similarity might be stronger than the potential influence of affective congruency, the present study only showed a significant priming type effect.

The N400 component yielded a significant effect of affective congruency, but not in the expected direction, only in Experiment 2 using emotional words as targets. Affectively congruent trials yielded more negative-going N400, compared to incongruent ones. That outcome was contrary to many previous affective priming studies, which reported more negative-going N400 components in incongruent trials. Earlier language and memory studies (Kounios and Holcomb, 1992; Kutas and Federmeier, 2000; Federmeier, 2007) first examined the N400 component, as its amplitude relies on the semantic relationship between a target and the context before it appears. Subsequently, N400 has also been explored in a few affective priming studies, as it reflects the mismatch between a prime and a target. For instance, when Zhang et al. (2010) presented participants with pictures as primes and words as targets, the results showed an evaluation incongruity effect on the N400 component with a latency range of 350–450 ms. Zhang et al. (2010) found the N400 component to be more negative-going to affectively incongruent than to affectively congruent trials. Krombholz et al. (2007) also reported such an effect when participants were asked to evaluated expressions with words as primes. On the one hand, some studies have found enhanced N400 effects on incongruent trials (Morris et al., 2003; Steinbeis and Koelsch, 2009; Eder et al., 2011). On the other hand, inverse N400 priming effect has also been observed in some circumstances (i.e., enhanced N400 amplitude on congruent trials compared to incongruent ones) in previous studies (Herring et al., 2011; Kissler and Koessler, 2011). How might one explain these contradictory findings?

In semantic priming studies, the reversed effect on N400 component could be explained by a center-surround inhibition mechanism (Bermeitinger et al., 2008). According to that theory, in order to increase the activation of the prime concept, the concepts surrounding the prime would become inhibited. Thus, impeded access to related targets then evoked enhanced N400 (i.e., congruent trials with related targets showed more negative-going amplitude). Consistent with the center-surround inhibition mechanism, Paulmann and Pell (2010) have reported a reversed N400 effect with faintly visible primes (SOA = 200 ms). They supposed that faintly visible primes can only weakly activate the concepts associated with the prime. To increase the contrast between the prime concept and others, the surrounding concepts were inhibited, leading to more complex activities. Applying this explanation to the present study, we assumed that the brief presentation of a geometric form only weakly activated its corresponding emotional representation, inhibiting the processing of a related target. Therefore, this emotionally related target became less accessible compared to an unrelated one and gave rise to more negative-going amplitude on congruent trials, resulting in the inverse N400 priming effect we obtained. Of course, there might be other possible reasons leading to this reversed effect. Further studies are needed to resolve this question. Moreover, although the N400 effect occurred at all scalp locations, we found more negative-going amplitude at anterior locations than at posterior areas (see **Figure 5**). Such a frontal N400 was reported by Krombholz et al. (2007) using word-expression pairs as stimuli. They inferred that the result reflected the involvement of frontal and prefrontal areas in cognitive and emotional processing.

Consistent with our expectation, a significant affective congruency effect on LPP occurred not only in Experiment1, but also in Experiment 2. Many studies (e.g., Werheid et al., 2005; Zhang et al., 2010, 2012; Herring et al., 2011; Hietanen and Astikainen, 2013) have demonstrated that the LPP component is modulated by affective congruency between the prime and the target. The affectively incongruent stimuli evoked larger amplitude LPP than affectively congruent ones and this LPP effect was considered as reflecting the increased attentional engagement under affectively incongruent conditions. Consistent with previous results, we also found a modulation of LPP by the affective congruency between the prime and the target, with enhanced LPP on incongruent trials compared to congruent ones. This result suggests that the emotional meaning of the circle and downward triangle can be activated automatically and can then influence the participants’ evaluation of targets on an electrophysiological level. Specifically, when prime and target have opposite valence, participants must expend more effort to evaluate targets. However, one thing to note here is that we obtained a significant LPP effect only on the POz electrode, but not on a wide range of brain areas in Experiment 1. This might have resulted from the different types of targets. The cognitive mechanisms involved need to be explored further in future investigations. In addition, the present study showed that negative targets were associated with a more positive-going LPP than positive targets. That finding was consistent with several studies (e.g., Ito et al., 1998; Wood and Kisley, 2006) but inconsistent with other many studies (e.g., Schapkin et al., 2000; Delplanque et al., 2004; Herbert et al., 2008) which found enhanced LPP responses to pleasant compared to unpleasant stimuli. In the present study, we inferred that enhanced LPP responses to negative targets, together with slower RTs to negative targets, probably reflected more cognitive processing demands for negative stimuli and greater difficulty in disengaging attention from negative stimuli compared to positive stimuli.

There are several limitations to our studies. One is the lack of an appropriate neutral prime condition. Although we included squares as shape primes in our experimental materials, the subjective evaluation results showed that the difference in valence between circle and square did not reach significance. Thus, squares are inadequate as neutral shape primes. Future research with demonstrably neutral prime stimuli is needed to help determine the source of affective congruency effects. Another limitation of the present study is a relative small sample size. Although our experiments had sufficient power to detect the influences of simple shapes, based on *p* values and effect size data provided in the result section, future studies with larger sample sizes would help support our conclusions. Finally, because no male participants took part in our experiments, our results are limited to female participants and the gender differences on the evaluation of simple geometric shapes cannot be investigated as well. Previous studies have showed some indications for gender differences in social cognition (see Pavlova, 2012). Further research including female and male participants is needed to explore the gender effect on the evaluation of shapes.

To sum up, using an affective priming paradigm, two experiments examined whether and how a circle and a downward triangle influence the affective processing of the faces and words. Although the expected behavioral effect of affective congruency did not emerge, we did find the hypothesized congruency effects at the electrophysiological level, mainly manifest as larger LPP component change in affectively incongruent trials compared to congruent trials. This LPP priming effect might suggest that the emotional meaning of a circle and a downward triangle is activated automatically and then impacts on the electrophysiological processing of subsequent stimuli. However, our findings are limited to females.

## Author Contributoins

YW co-designed the experiment, collected, and analyzed the data and co-wrote the text. QZ co-designed the experiment, advised on many aspects of the research and co-wrote the text.

## Conflict of Interest Statement

The authors declare that the research was conducted in the absence of any commercial or financial relationships that could be construed as a potential conflict of interest.
